# OLDER ADULTS HAVE DIFFERENTIAL RESPONSE PATTERNS TO TWO COMMON SUICIDE ASSESSMENT MEASURES

**DOI:** 10.1093/geroni/igae098.1835

**Published:** 2024-12-31

**Authors:** Fatema Colombowala, Emily Bower

**Affiliations:** Pacific University, Hillsboro, Oregon, United States; Pacific University, Hillsboro, Oregon, United States

## Abstract

An alarming number of older adults die by suicide each year. Older adults exhibit lower rates of suicidal ideation (SI) yet higher rates of suicide deaths compared to younger adults, so improving risk detection is imperative. Our aim was to compare response patterns to two common, validated measures for assessing SI in older adults at higher risk for suicide. We hypothesized there would be a positive relationship between responses to the two assessments for SI and death ideation (DI). We also expected variation in response patterns among some participants, and examined these exploratorally. Preliminary analyses included 41 participants (M=70 years, 78% female) with subjective cognitive decline who completed the Geriatric Suicide Ideation Scale (GSIS) and the Columbia Suicide Severity Rating Scale (CSSRS) as part of a larger study. The GSIS is a self-report measure developed with and for use with older adults that includes SI and DI subscales. The CSSRS is a clinician-administered assessment of SI severity. 62% endorsed lifetime SI and 12% endorsed past-month SI. CSSRS lifetime and past-month SI scores were strongly correlated with the GSIS SI subscale scores (r’s(39)=.5-.6, p’s <.001). However, CSSRS past-month DI scores were not correlated with GSIS DI subscale scores (r(39)=.09, p >.05). Furthermore, we observed endorsement of GSIS DI items among participants who denied DI on the CSSRS. Differential response patterns highlight the need for meaningful measures and/or adaptations of existing measure to accurately capture suicide risk.

